# Retraction statement: MicroRNA‐519a promotes proliferation and inhibits apoptosis of hepatocellular carcinoma cells by targeting FOXF2

**DOI:** 10.1002/2211-5463.13530

**Published:** 2022-12-12

https://doi.org/10.1016/j.fob.2015.10.009

The above article published online on 10 November 2015 in Wiley Online Library (wileyonlinelibrary.com) has been retracted by agreement between the authors, the journal's Editor‐in‐Chief Miguel De La Rosa, FEBS Press, and John Wiley and Sons Ltd. The retraction has been agreed upon following an investigation based on allegations raised by a third party, which revealed that some images in this article are inappropriate duplications of images from a previously published article [1]. Thus, the editors consider the conclusions of this manuscript substantially compromised.
